# Performance in play: stimulant exposure and broader substance-use vulnerability in mass-participation youth esports

**DOI:** 10.3389/fpubh.2026.1988864

**Published:** 2026-10-07

**Authors:** Rui-Yan Sun, Si-Yuan Zhao

**Affiliations:** 1 Hero Esports, Inc., Shanghai, China; 2 Faculty of Humanities and Arts, Macau University of Science and Technology, Taipa, Macao SAR, China

**Keywords:** adolescents, caffeine, energy drinks, sleep, sugar, video gaming

## Abstract

Youth esports now extends far beyond professional competition, encompassing ranked online play, school clubs, amateur events, livestreaming, and digitally mediated peer communities. These settings expose adolescents to energy drinks and other stimulant-containing products in contexts where alertness, concentration, and prolonged play are valued. Energy drinks merit particular attention because their prominence in gaming culture combines caffeine exposure with, for many products, substantial added sugar. Potential consequences include disrupted sleep, acute stimulant effects, and adverse nutritional and metabolic profiles. A further concern is behavioral: repeated consumption for gaming-related fatigue or concentration may foster expectations that psychoactive products are useful tools for performance. Research on nonmedical prescription-stimulant use among adolescents demonstrates a comparable performance-oriented motive, while longitudinal studies link energy-drink use with later tobacco, alcohol, and other substance involvement. These associations remain non-causal and have not been established specifically in recreational esports. This Perspective frames mass-participation youth esports as an emerging setting for early public-health intervention. It proposes action across marketing and sponsorship, schools and community programs, beverage and product policy, families and healthcare, and longitudinal surveillance, while emphasizing the need to establish prevalence, temporal ordering, and high-risk patterns directly in nonprofessional adolescent participants across different social and regulatory settings.

## 1 Introduction

Competitive video gaming has become part of the everyday digital environment of many adolescents. Ranked multiplayer systems allow competition to occur continuously through personal computers and mobile devices, while school programs, community clubs, amateur leagues, open online tournaments, livestreams, and social media extend competitive gaming beyond the game itself. In a population-based study of 70,695 Norwegian adolescents aged 16–18 years, approximately 10% reported some esports training or competition and 4.7% met stricter criteria for active esports participation (1). School-based research similarly shows that esports can function as an extracurricular and recreational activity rather than primarily as preparation for a professional career (2, 3). Together, these forms of participation constitute a broad nonprofessional layer of competitive gaming that is increasingly embedded in adolescent daily life. We use mass-participation youth esports to describe this layer, including repeated ranked play, school and community esports, amateur and open online competition, and the livestreaming, social-media, and peer environments that surround them.

Within this wider participation environment, substance exposure is likely to occur differently from that described in professional esports. Discussions of substance use in elite competition have largely emphasized prohibited substances and competition integrity. Young recreational participants, however, encounter caffeinated beverages, energy drinks, and gaming-oriented supplements through ordinary retail, online marketing, livestreams, and peer networks. These products may become associated with the practical demands of competitive play when sessions extend late into the evening or when players perceive sustained concentration, reaction speed, and resistance to fatigue as important to performance. Their accessibility allows stimulant use for gaming-related purposes to develop within ordinary adolescent routines, well outside formal competitive or sporting structures.

These conditions raise a broader public-health question: can mass-participation esports become an environment in which substance-assisted performance is normalized among young people, and should prevention begin before such patterns become established or progress toward more harmful forms of stimulant or substance use? This Perspective examines the evidence relevant to that possibility and considers how prevention can be incorporated into the commercial, social, educational, and healthcare environments surrounding youth participation.

## 2 Competitive gaming as a setting for stimulant exposure

The commercial environment surrounding esports gives energy drinks unusual visibility within gaming culture. Long et al. (4) identified 497 food and beverage sponsorship relationships across major esports events, leagues, teams, and players; energy-drink brands accounted for 181 relationships, or 36.4% of the total. Although these sponsorships involved prominent esports properties, their audiences extend well beyond professional competitors through streams, social-media accounts, branded content, and creator communities. Livestreaming further reduces the separation between entertainment and promotion. Evans et al. (5) analyzed *Fortnite* streams from Twitch creators likely to attract adolescent audiences and found food and beverage cues occupying substantial portions of viewing time, with advertising disclosure attached to only a small proportion of those cues. Product exposure can consequently occur while a young person is watching gameplay, interacting with chat, following a favored creator, or learning strategies for a game.

Evidence from adolescents provides a more direct connection between gaming and energy-drink exposure. Yang et al. (6) surveyed 2,613 seventh-grade students in Taiwan; 75% reported seeing energy-drink advertising online during the previous year and almost 18% had consumed energy drinks. Excessive gaming and greater exposure to online energy-drink marketing were independently associated with consumption. The cross-sectional design does not determine whether gaming exposure led to beverage use, but the findings show that gaming behavior, digital marketing, and energy-drink consumption already coexist within an adolescent population.

Marketing also shapes what the product represents. Park et al. (7) describe caffeine and energy drinks as embedded in gaming culture through associations with alertness, achievement, social belonging, and gamer identity. A beverage repeatedly displayed alongside competitive play may acquire performance relevance even when an advertisement makes no formal efficacy claim. The product is encountered in a context in which concentration and persistence matter to the player. The belief is reinforced by a developing literature on caffeine and gaming performance. Huang et al. (8) identified 18 supplement protocols across 13 randomized esports trials, 14 of which contained caffeine. Some studies reported changes in reaction time, attention, mood, or gaming performance. These adult and predominantly competitive-player studies cannot establish benefits for adolescents. They nevertheless help explain why caffeine can plausibly be presented to consumers as useful for gaming.

The relevance of energy drinks to youth esports therefore arises from more than their caffeine content. They are accessible consumer products whose commercial presentation, cultural visibility, and pharmacological effects can all support a performance-oriented interpretation. Their health implications become particularly important when consumption occurs during prolonged or late gaming.

## 3 Energy drinks: a dual stimulant and metabolic exposure

Current pediatric guidance provides a clear reference point. Consensus recommendations developed with representatives from major US nutrition, pediatric dentistry, pediatrics, and cardiovascular organizations advise children and adolescents aged 5–18 years to avoid energy drinks and other beverages containing caffeine or stimulants (9). Gaming culture can expose young people to a competing message in which the same products are associated with wakefulness and performance.

Sleep is one of the most plausible pathways through which gaming-related caffeine use can become self-reinforcing. Adolescents aged 13–18 years are generally recommended to obtain 8–10 h of sleep per night (10). Evening gaming already reduces available sleep time when sessions extend toward bedtime. Carneiro et al. (11), synthesizing 18 studies involving 188,322 adolescents across 13 countries, found energy-drink consumption consistently associated with shorter sleep duration, poorer sleep quality, and greater daytime dysfunction.

The timing of consumption is especially relevant. Caffeine used to maintain concentration during a late gaming session may delay sleep or impair recovery, leaving greater fatigue the following day. Repeating this pattern can make stimulant use part of the response to insufficient sleep. Prospective esports-specific evidence is currently lacking, but this interaction between gaming schedules, fatigue, and caffeine is sufficiently plausible to warrant direct investigation among recreational competitors.

Energy drinks also create a nutritional concern that is separate from their stimulant effects. Ajibo et al. (12) reviewed 57 studies involving children and young people and reported associations between energy-drink consumption and multiple adverse physical and behavioral outcomes, including insulin resistance. Many products contain substantial added sugar, placing them within the wider problem of sugar-sweetened beverage consumption. Sugar-sweetened beverages contribute to excessive energy intake and childhood overweight and obesity, supporting population-level approaches such as taxation and healthier school food environments (13).

The gaming context is relevant because a sugar-sweetened energy drink may be consumed repeatedly during an activity with little energetic demand and for reasons unrelated to nutritional need. Evidence does not currently demonstrate that esports amplifies the metabolic effects of these beverages, but routine gaming-related consumption adds an avoidable source of liquid sugar and energy. Sugar-free formulations remove this particular burden while retaining caffeine and other stimulant exposures. They should not automatically acquire a health halo: human experimental research has raised questions about sweetener-specific metabolic effects, although pediatric evidence remains limited (14).

Acute adverse effects provide another consideration. Li et al. (15) identified cardiovascular and neuropsychiatric events among published pediatric cases following energy-drink consumption, with pre-existing conditions or additional triggers present in many reports. Severe events are uncommon and case reports cannot estimate population risk, but symptoms such as palpitations, anxiety, tremor, and sleep disturbance are relevant when caffeine exposure occurs without clinical supervision.

The longer-term behavioral issue begins when stimulant use acquires a consistent purpose. An adolescent who repeatedly consumes energy drinks to remain awake, maintain concentration, or prevent perceived deterioration in gaming performance is learning to use a psychoactive substance instrumentally. Similar motives are already documented in another area of adolescent stimulant use.

## 4 Performance-motivated stimulant use and broader substance-use vulnerability

Nonmedical prescription-stimulant use among US adolescents provides an informative analogue because perceived cognitive performance is a documented motive for use. Teter et al. (16) analyzed 15,098 US high-school seniors. Among students reporting past-year nonmedical prescription-stimulant use, 48.3% reported studying as either their sole motive or one of several motives. These users did not demonstrate superior academic performance and had greater involvement in binge drinking, cigarette smoking, marijuana use, and nonmedical opioid use. The parallel with competitive gaming concerns the role attributed to the stimulant. Academic misuse can be motivated by the desire to study longer or concentrate more effectively; caffeine may be used during gaming to maintain wakefulness or perceived performance. Prescription stimulants differ greatly from energy drinks in pharmacology, regulation, and risk, so this analogy should not be interpreted as a sequence of escalation.

Energy-drink studies nevertheless raise a broader question about subsequent substance involvement. Yasuma et al. (17) reviewed prospective adolescent cohorts and found associations between energy-drink consumption and later alcohol, tobacco, cannabis, prescription-stimulant, and analgesic use. Galimov et al. (18) followed 3,071 adolescents aged 9–17 years who had not previously used the substances examined and found baseline energy-drink consumption associated with initiation of cigarettes, e-cigarettes, hookah, and alcohol during the following 12 months.

These associations have several possible explanations. Energy-drink users may differ in sensation seeking, peer networks, parental monitoring, sleep patterns, or pre-existing propensity toward risk behavior. Ajibo et al. (12) similarly found energy-drink use clustering with smoking, alcohol use, binge drinking, and other substance-related behaviors across the youth literature. Current evidence therefore supports energy-drink consumption as a potential marker of broader behavioral vulnerability, although the direction and mechanisms of this relationship remain uncertain. Figure 1 integrates these observations into the proposed pathway linking the mass-participation esports environment, performance-oriented stimulant use, health consequences, and broader substance-use vulnerability.

**Figure 1:**
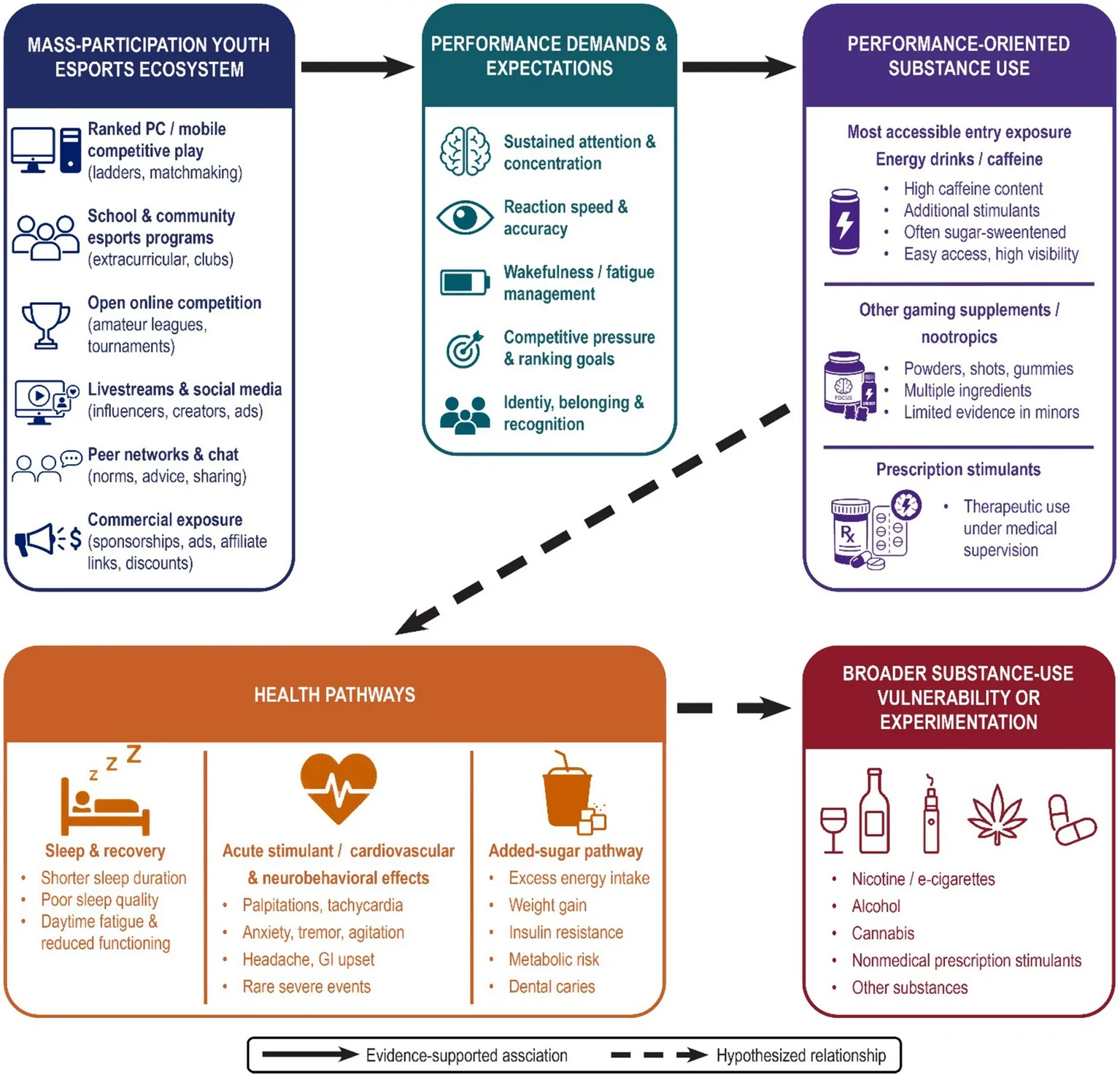
Proposed pathways linking mass-participation youth esports with performance-oriented stimulant use and related health risks.

Mass-participation esports may provide a setting in which these behavioral expectations are particularly relevant. Repeated use of energy drinks for gaming may strengthen expectations that fatigue or concentration can be managed through stimulant use. Such expectations may alter later receptivity to supplements, nicotine, nonmedical prescription stimulants, alcohol, or other substances. Because these expectations can develop well before clinically apparent misuse, prevention has to address the environments in which products are first encountered and given meaning.

## 5 Public-health prevention in the mass-participation environment

The preceding evidence places the public-health concern upstream of clinically recognizable stimulant misuse. Performance-related meanings can develop while energy drinks and other products are still encountered through ordinary gaming, marketing, peer interaction, and recreational competition. Prevention can therefore operate across several levels of this environment, from commercial exposure and product policy to organized youth esports, family and clinical practice, and population surveillance. Table 1 summarizes these intervention domains and their corresponding child-health rationale.

**Table 1:** Public-health actions for stimulant-related exposure in mass-participation youth esports.

Policy domain	Application to youth esports	Child-health rationale
Commercial marketing and sponsorship	Limit energy-drink and focus-oriented supplement sponsorship in school and youth esports; restrict performance-linked promotion directed toward minors	Reduce repeated associations between stimulant products, gaming identity, and performance
Digital platforms	Strengthen age-sensitive advertising controls and disclosure of sponsored or affiliate content	Extend child-marketing protections into livestreaming and social-media environments
Beverage and product policy	Clearly disclose caffeine and added sugar; include qualifying sugar-sweetened energy drinks within relevant fiscal and nutrition policies; consider age-related sales measures according to jurisdiction	Reduce exposure and improve product transparency
School and community esports	Make water the default beverage; exclude energy-drink promotion; avoid unnecessarily late scheduling; establish symptom and referral procedures	Reduce institutional endorsement and interaction between stimulant use and sleep loss
Family and clinical practice	Ask about product, dose, timing, motive, sleep, supplements, and nonmedical medication use in relation to gaming	Detect emerging performance-motivated use while protecting appropriate medical treatment
Surveillance and research	Measure gaming intensity, commercial exposure, caffeine dose and timing, beverage formulation, sleep, motives, and other substance use longitudinally	Establish prevalence, temporal relationships, and high-risk patterns

The first opportunities arise before consumption itself, through the commercial and product environments that shape what young players encounter and how these products are presented. Energy-drink sponsorship and influencer promotion are already well established within gaming media, while adolescent evidence links online marketing exposure with energy-drink consumption (4, 6). Youth-facing esports should therefore be included within the broader public-health discussion surrounding the marketing of caffeinated and nutritionally poor beverages to minors. School esports, community youth programs, and competitions explicitly directed toward minors have particularly strong grounds for excluding energy-drink sponsorship and performance-linked promotion. Livestreaming and social-media platforms could strengthen age-sensitive advertising controls and require clear disclosure of paid promotion, affiliate marketing, and sponsored creator content.

Existing beverage policies provide mechanisms through which these principles could be applied. Rostami et al. (19) documented energy-drink policies across 73 countries and territories, including taxation, school restrictions, sales controls, labeling requirements, and marketing restrictions. Several of these approaches can be extended to digital gaming environments, with implementation reflecting national regulation and patterns of youth access. Sugar-sweetened energy drinks can also be addressed through broader beverage taxation and school procurement policies where these mechanisms already operate. Product labels should present caffeine and added-sugar content in forms that young consumers and parents can readily interpret.

Schools and community esports programs provide a more immediate setting for prevention. Water can remain the default beverage during organized activities, and programs can avoid distributing or promoting energy drinks as performance aids. Scheduling also deserves attention because unnecessarily late competition may increase the appeal of stimulant use. Staff guidance should distinguish energy drinks from sports drinks and establish procedures for responding to palpitations, dizziness, marked anxiety, or severe sleep disturbance. Coaches and program staff should not recommend supplements or medication for gaming performance.

Family guidance and clinical screening become more relevant once use is occurring. Information about timing and motive can distinguish occasional consumption from a pattern in which caffeine is routinely used to extend play, maintain concentration, or compensate for fatigue. Clinical histories can include energy drinks, supplements, and medications used specifically to remain awake or concentrate during gaming. For adolescents receiving stimulant therapy for attention-deficit/hyperactivity disorder, assessment should remain focused on therapeutic response, adherence, adverse effects, and any diversion or unsupervised changes in use.

Surveillance is necessary to establish how often these patterns occur in mass-participation youth esports. Studies should distinguish competitive participation from gaming in general and measure the timing and intensity of play, caffeine dose, beverage formulation, reasons for consumption, sleep, supplement use, commercial exposure, and subsequent substance-related behaviors. Prospective designs are particularly important for determining whether gaming-related stimulant use precedes sleep disruption or broader substance involvement, rather than simply co-occurring with them. Cross-national research would also help clarify how differences in advertising regulation, energy-drink availability, school policies, and prescribing practices modify these relationships.

## 6 Discussion

Mass-participation youth esports warrants attention as an emerging environment in which stimulant use can become connected with perceived performance. Energy drinks are currently the most visible product through which this connection can develop: they are accessible to adolescents, heavily represented within gaming culture, and pharmacologically suited to the immediate demands of late or prolonged play. When consumption becomes a routine response to fatigue or concentration difficulty, the behavior has public-health relevance even in the absence of prohibited substances or clinically recognized misuse.

The possibility of broader substance-use vulnerability gives this issue greater importance but also requires caution. Adolescent research demonstrates that stimulants can be used instrumentally for perceived cognitive performance, and prospective energy-drink studies show associations with subsequent alcohol, tobacco, and other substance use. Esports-specific evidence has yet to establish whether performance-motivated caffeine use contributes to such trajectories. Shared behavioral characteristics and social environments may account for much of the association. The pathway proposed here should therefore guide longitudinal research.

This uncertainty does not remove the opportunity for prevention. Most of the proposed measures address exposures that already exist and are consistent with established child-health principles: reducing youth-directed marketing of stimulant beverages, limiting institutional promotion in schools and community programs, protecting sleep through sensible scheduling, improving beverage information, and identifying performance-motivated use when it emerges. Applying these principles to competitive gaming recognizes that health behavior is increasingly shaped within digital environments as well as physical ones.

Mass-participation esports will remain part of adolescent recreation and social life. Public health can engage with that environment without pathologizing gaming or treating young players as prospective substance users. The immediate task is to prevent energy drinks and related products from becoming routine tools for sustaining play and to determine whether adolescents who adopt that pattern carry greater substance-related risk over time. Addressing the issue at the recreational level brings prevention closer to the population in which these habits are most likely to begin.

## Data Availability

The original contributions presented in the study are included in the article/supplementary material, further inquiries can be directed to the corresponding author.
